# Awareness, Attitudes, and Utilization of Counseling Services Among Students at the University of Sharjah, UAE: A Cross-Sectional Study

**DOI:** 10.7759/cureus.104598

**Published:** 2026-03-03

**Authors:** Yasmeen S Rahmi, Sara H Abdelrasool, Abdul Rahman A Sheiko, Toleen G Tuhul, Maha J Almheiri, Abdulla J Sawalha, Hiba J Barqawi, Amal Hussein

**Affiliations:** 1 Clinical Sciences, College of Medicine, University of Sharjah, Sharjah, ARE; 2 Research Institute of Medical and Health Sciences, University of Sharjah, Sharjah, ARE; 3 Family and Community Medicine and Behavioral Sciences, College of Medicine, University of Sharjah, Sharjah, ARE

**Keywords:** academic stress, counseling utilization, help seeking behavior, mental health services, mental health stigma, student mental health, university counseling, university students

## Abstract

Background

University students experience high levels of academic and psychological stress, yet counseling services remain underutilized, particularly in Middle Eastern settings, where stigma and limited awareness may affect help-seeking. Although studies in the United Arab Emirates (UAE) have examined general attitudes toward professional psychological help-seeking, institution-specific evidence on students’ awareness of campus counseling services, perceived confidentiality, and actual utilization remains limited. In particular, data describing these factors among students at the University of Sharjah (UOS) are lacking. This study assessed awareness, attitudes, and practices related to counseling services among students at UOS.

Methods

A descriptive cross-sectional survey was conducted among undergraduate students at UOS between March and June 2023 using a bilingual, self-administered questionnaire. Convenience sampling yielded 491 participants. Data were analyzed using descriptive statistics and Chi-square tests (p ≤ 0.05). As a non-probability convenience sample, the findings may be subject to selection bias and limited generalizability.

Results

Most participants were aged 18-24 years (462, 94.1%) and female (354, 72.1%). Overall, 307 (62.5%) were unaware of university counseling services, and 322 (65.6%) reported confidentiality concerns. High academic stress was common, with 389 (79.2%) reporting difficulty managing stress and time during examinations. Despite this, only 34 (8.7%) of stressed students used university counseling services. Friends (187, 38.0%) and family (167, 34.0%) were the main support sources. Major barriers included lack of awareness of service location (218, 44.4%), reluctance to share personal information (199, 40.5%), and uncertainty about access (177, 36.1%). Female gender was associated with greater stress and study difficulty (302, 85.3% vs 87, 63.5%; χ² = 5.64, p = 0.018, Cramér’s V = 0.11), with females showing higher odds of stress than males (OR = 3.33, 95% CI: 2.08-5.33). Face-to-face (398, 81.1%) and individual counseling (445, 90.6%) were preferred.

Conclusions

A substantial gap exists between students’ mental health needs and counseling utilization at UOS. Limited awareness, stigma-related concerns, and accessibility barriers contribute to underuse. Enhancing outreach and improving the visibility of and trust in services may increase counseling engagement among UAE university students.

## Introduction

University life represents a critical developmental period characterized by academic demands, social transitions, and increasing personal responsibility. During this stage, students experience multiple stressors that increase vulnerability to psychological distress, with young adults disproportionately affected by mental health conditions globally [1]. University counseling services, therefore, play an essential role in supporting students’ mental health and overall functioning. However, counseling services remain underutilized, particularly in non-Western contexts, due to a lack of awareness, stigma, negative attitudes, and cultural beliefs surrounding professional psychological help-seeking [2-4].

Mental health disorders constitute a major global public health concern. According to the World Health Organization (WHO), more than a billion people worldwide live with a mental health condition, contributing substantially to global disease burden [1]. Depression and anxiety disorders are leading causes of disability, particularly among young adults, the primary age group represented in university populations [1]. Mental health has also been recognized as an international policy priority, with global initiatives emphasizing the need to strengthen access to psychological services [5]. Studies in higher-education settings further indicate a high prevalence of mental health conditions among university students and increasing demand for accessible campus-based psychological support [6,7].

Within the Middle East and North Africa (MENA) region, university students experience comparable or higher levels of psychological distress, yet professional help-seeking remains limited. Cultural norms emphasizing family cohesion, privacy, and religious coping often lead individuals to rely on informal support networks rather than professional psychological services [2,3]. Studies across regional contexts, including Turkey and neighboring Middle Eastern countries such as Jordan and Saudi Arabia, demonstrate that attitudes toward seeking professional psychological help are strongly shaped by stigma, perceived social judgment, and cultural beliefs regarding self-reliance and emotional disclosure [4,8-10]. Similar barriers related to awareness and cultural perceptions have also been reported across MENA populations, further indicating that sociocultural factors play a central role in counseling utilization [11].

In the United Arab Emirates (UAE), mental health has become an emerging national priority, reflected in the launch of the National Policy for the Promotion of Mental Health in 2017 and ongoing initiatives aimed at strengthening community-based and digital mental health services. Nevertheless, help-seeking behaviors remain influenced by sociocultural attitudes. Studies among Emirati university students have reported reluctance to disclose personal problems to professionals due to societal norms that may associate help-seeking with weakness or loss of social standing [2,3]. Approximately 45% of UAE university students were previously reported to be unaware of available counseling services, and many preferred support from family or friends during psychological distress [2]. Concerns regarding confidentiality, stigma, and trust in institutional services have also been consistently identified as barriers within the UAE context [3].

Despite increasing national attention to mental health, institution-specific evidence regarding awareness and utilization of university counseling services within the UAE remains limited, particularly outside major metropolitan institutions. The University of Sharjah (UOS) is one of the largest higher-education institutions in the Northern Emirates, hosting a diverse student population across multiple academic disciplines [12]. However, data on students’ awareness, attitudes, and practices related to counseling service utilization at UOS remain scarce. Understanding these factors is essential for guiding culturally appropriate mental health promotion strategies and improving service accessibility within university settings in the UAE.

This study, therefore, aims to assess the awareness, attitudes, and practices of students at UOS regarding the utilization of university counseling services. Secondary objectives were to explore students’ preferences for counseling delivery mode and format, identify perceived barriers to counseling utilization, and examine student characteristics associated with counseling awareness and utilization.

To address these objectives, the study examined the following research questions: (i) What is the level of awareness of university counseling services among UOS students? (ii) What are students’ attitudes toward counseling services and their perceived importance? (iii) What are the patterns of counseling utilization among UOS students? (iv) What barriers influence students’ willingness to seek university counseling services? (v) What are students’ preferences regarding counseling delivery mode and format? (vi) Which student characteristics are associated with counseling awareness and utilization?

## Materials and methods

Study design, setting, and population

A descriptive, cross-sectional study was conducted to assess the awareness, attitudes, and practices of students at UOS regarding the utilization of university counseling services. The target population consisted of undergraduate students enrolled at UOS during the study period. Inclusion criteria included students aged 17 years and above who were currently registered at the university and able to read and understand either English and/or Arabic, as the questionnaire was available in these two languages (see Appendix A). Students currently receiving medical treatment for psychological or psychiatric disorders, and individuals who were relatives or close acquaintances of university counselors, were excluded from the study to avoid potential confounding related to prior clinical exposure and familiarity with mental health services; however, treatment status was not directly assessed in the questionnaire, and therefore no exclusions were applied during recruitment.

Non-probability, convenience sampling was used to recruit participants. The questionnaire was distributed electronically through official university email lists, student WhatsApp groups, and social media platforms. As the survey link was disseminated through these channels, the total number of students who received the invitation could not be determined; therefore, a response rate could not be calculated. Data collection was conducted between March 2023 and June 1, 2023. A minimum sample size of 385 participants was calculated based on a 5% margin of error and an assumed prevalence of 50%, using Cochran’s formula for estimating a single population proportion: $n = \frac{Z^2 \, p (1 - p)}{e^2}$, where n represents the sample size, p represents the expected prevalence, Z corresponds to the standard normal distribution value at a 95% confidence level (1.96), and e represents the margin of error. A total of 491 students completed the questionnaire, exceeding the minimum required sample size.

Ethical considerations

The study was conducted in accordance with the Declaration of Helsinki and approved by the Ethics Committee of the UOS (REC-23-02-18-13-S). Written informed consent was obtained electronically from all participants prior to completing the questionnaire. Although the study was anonymous, confidentiality was maintained by storing all data in an encrypted, password-protected drive, accessible only to the research team and supervisor.

Questionnaire development

A self-administered structured questionnaire was developed after reviewing relevant literature and previously published studies examining awareness and attitudes toward university counseling services. The questionnaire was originally developed in English and subsequently translated into Arabic to ensure inclusivity and accessibility. The Arabic version was reviewed by bilingual members of the research team and the academic supervisor to ensure linguistic accuracy, conceptual equivalence, and cultural appropriateness. Minor wording adjustments were made through consensus prior to pilot testing.

The final questionnaire consisted of 25 questions divided into four sections: demographics (seven questions), awareness of counseling services (five questions), academic performance and stress (four questions), and attitudes and practices toward counseling services (nine questions). The questionnaire included multiple-choice questions and Likert-scale items. Likert-scale items were analyzed descriptively as ordinal categorical variables without composite scoring.

As the questionnaire was developed specifically for this study and was not based on a previously validated standardized scale, formal psychometric validation was not performed. Because the questionnaire primarily comprised single-item measures and categorical variables, rather than multi-item psychometric scales, internal consistency reliability indices such as Cronbach’s alpha were not calculated. Content validity was ensured through review by the research team and the academic supervisor to confirm relevance and clarity of items. The questionnaire was piloted among 15 students to assess clarity, comprehension, and completion time. Minor modifications were made based on pilot feedback prior to final distribution.

Before data collection, the research team agreed on standardized procedures for questionnaire distribution and participant communication to ensure consistency in data collection.

Data collection and analysis

During data collection, the questionnaire was distributed electronically through official university email lists, student WhatsApp groups, and social media platforms. Participants completed the questionnaire voluntarily using their personal devices. To minimize duplicate responses, the survey was restricted to one response per institutional email account, and participation was limited to UOS student email addresses. The study aims and objectives were clearly explained at the beginning of the survey through an electronic information sheet. Completion of the questionnaire was considered implied consent to participate in the study. No names or identifying information were collected to ensure anonymity. Confidentiality was maintained, and collected data were stored in an encrypted, password-protected database, accessible only to the research team.

Data analysis was conducted using IBM SPSS Statistics for Windows, Version 26 (Released 2018; IBM Corp., Armonk, NY, USA). Demographic variables included age, gender, ethnicity, college, academic program, and employment status. Academic stress was operationalized using the item assessing difficulty managing stress and time during examination periods (yes/no). Categorical variables were summarized using frequencies and percentages. Missing responses were minimal and handled by reporting valid percentages for each item.

Associations between categorical variables were examined using the Chi-square (χ²) test. Valid percentages were reported to account for any missing data. Since the majority of variables were categorical and descriptive in nature, scoring systems or composite indices were not generated. A p-value of ≤0.05 was considered statistically significant. Because the study was primarily descriptive, and the number of outcome events for some variables (e.g., counseling utilization) was limited, multivariable regression modeling was not performed to avoid unstable estimates. Additionally, because multiple bivariate comparisons were performed in an exploratory context, no formal adjustment for multiple testing was applied, and results were interpreted cautiously.

The methodological quality and reporting of this study were guided by the Strengthening the Reporting of Observational Studies in Epidemiology statement for cross-sectional studies. 

## Results

Participant demographics

A total of 491 students from UOS participated in the study. The majority were aged 18-24 years, 462 (94.1%), and female, 354 (72.1%). Most participants were undergraduate students, 468 (95.3%), and not employed at the time of the study, 454 (92.5%). With respect to nationality, the largest proportion were students from other Arab nationalities, 292 (59.5%), followed by Emirati students, 155 (31.6%). Slightly more than half of the participants were enrolled in non-health-related colleges, 275 (56.0%), while students from health-related colleges (Medicine and Health Sciences) represented 216 (44.0%) of the sample (Table 1).

**Table 1:** Sociodemographic characteristics of participants (N = 491).

Variable	Category	n (%)
Age group	18-24 years	462 (94.1)
	≤17 years	18 (3.7)
	≥25 years	11 (2.2)
Gender	Female	354 (72.1)
	Male	137 (27.9)
Ethnicity	Emirati	155 (31.6)
	Other Arab nationalities	292 (59.5)
	Non-Arab	44 (9.0)
College	Medicine	158 (32.2)
	Engineering and Informatics Programs	69 (14.1)
	Health Sciences	58 (11.8)
	Other colleges: Dental, Pharmacy, Sciences, Islamic Studies (Sharia), Fine Arts, Communication, Law, and Business programs	206 (41.9)
Program	Undergraduate	468 (95.3)
	Higher diploma / Master’s / PhD	23 (4.7)
Employment status	Not working	454 (92.5)
	Currently working	37 (7.5)

Awareness and knowledge of counseling services

More than half of the students, 307 (62.5%), reported that they were not aware that the university provides counseling services. Among students who were aware of the counseling services, friends were the most frequently reported source of awareness, 75 (15.3%), followed by faculty or staff members, 40 (8.1%), the university website, 37 (7.5%), and social media platforms, 33 (6.6%). Despite this, 393 (80.0%) of those reported being aware of the counseling department’s location and contact details. Concerns regarding confidentiality were common, with 322 (65.6%) of students expressing concern when considering seeking counseling services.

Students demonstrated variable knowledge regarding the services provided by the counseling department. Approximately one-quarter of respondents identified academic advising, 123 (25.0%), and general problem-solving support, 123 (25.0%), as services offered by the department. Fewer students recognized more specialized services, such as referral to specialists, 76 (15.4%), group therapy, 66 (13.5%), or family counseling, 47 (9.6%). A small proportion of participants incorrectly believed that counselors could prescribe medication, 24 (4.8%), indicating gaps in understanding of the scope of counseling services (Table 2).

**Table 2:** Awareness and attitudes of UOS students toward counseling services. * Multiple responses allowed; therefore, percentages may not total 100%.

Variable	Category	n (%)
Awareness of the University of Sharjah (UOS) counseling services	Aware	184 (37.5)
	Not aware	307 (62.5)
Source of awareness	Friends	75 (15.3)
	Faculty/Staff	40 (8.1)
	UOS website	37 (7.5)
	Social media	33 (6.6)
	Not aware	307 (62.5)
Awareness of the counseling department location/contact	Aware	393 (80.0)
	Not aware	98 (20.0)
Concern about confidentiality	Concerned	322 (65.6)
	Not concerned	169 (34.4)
Knowledge of counseling services offered*	Academic advice	123 (25.0)
	General problem-solving support	123 (25.0)
	Referral to a specialist	76 (15.4)
	Group therapy	66 (13.5)
	Family counseling	47 (9.6)
	Gender-segregated services	33 (6.6)
	Prescription of medication	24 (4.8)
Perceived importance of counseling	Extremely important	80 (16.3)
	Moderately important	212 (43.2)
	Slightly important	141 (28.7)
	Not important at all	58 (11.8)

Attitudes and practices toward counseling services

Regarding personal priorities, mental health was the most frequently reported top priority, 210 (42.8%), followed by studies, 162 (33.0%), and friends and family, 83 (16.9%).

Nearly half of the students reported dissatisfaction with their recent grades, 255 (51.9%). A substantial proportion reported difficulty finding suitable study methods, 330 (67.2%), and difficulty managing stress and time during examination periods, 389 (79.2%).

Help-seeking behavior varied, with 193 (39.3%) actively seeking help when facing problems, 141 (28.7%) expressing a desire to seek help but not currently doing so, and 157 (32.0%) reporting that they did not seek help. Friends, 187 (38.0%), and family members, 167 (34.0%), were the most common sources of support, whereas 69 (14.0%) of students reported not seeking help from anyone. Very few students sought help from mental health professionals or university personnel.

**Figure 1:**
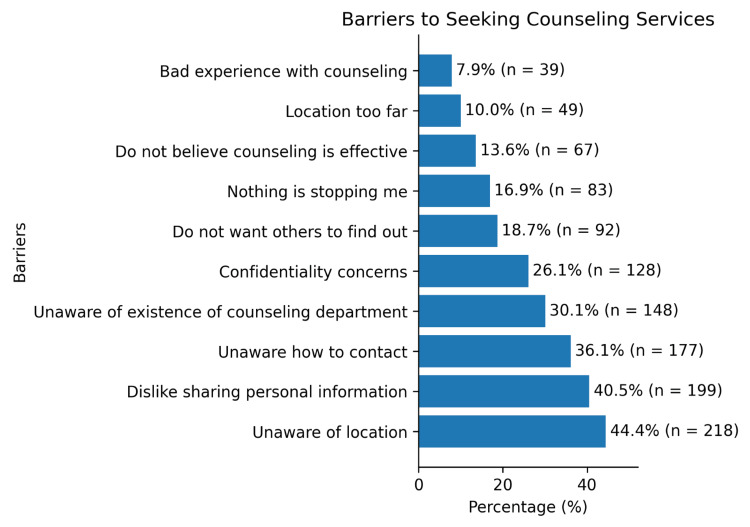
Barriers to seeking counseling services among the University of Sharjah (UOS) students (N = 491). Participants could select more than one barrier; therefore, percentages may not total 100%.

The most frequently reported barrier to utilizing the counseling department was lack of awareness of the counseling department’s location, 218 (44.4%), followed by reluctance to share personal information, 199 (40.5%), and uncertainty about how to contact the department, 177 (36.1%) (Figure 1). Most students rated seeking counseling services as moderately important, 212 (43.2%). Only 50 (10.2%) students reported having tried counseling services within the UOS, whereas 75 (15.3%) had sought counseling outside the university; most participants had not utilized counseling either within UOS, 441 (89.8%), or externally, 416 (84.7%). Face-to-face counseling was the preferred mode of service delivery, 398 (81.1%), and individual counseling was strongly preferred over group counseling, 445 (90.6%). Regarding communication preferences, more than half of the participants preferred contacting the counseling department through in-person visits, 254 (51.7%), followed by email, 136 (27.7%), and phone calls, 101 (20.6%) (Table 3).

**Table 3:** Counseling-related practices.

Variable	Category	n (%)
Satisfaction with grades	Satisfied	236 (48.1)
	Not satisfied	255 (51.9)
Student's top priority	Mental health	210 (42.8)
	Studies	162 (33.0)
	Friends & family	83 (16.9)
	Others	36 (7.4)
Difficulty finding a suitable study method	Yes	330 (67.2)
	No	161 (32.8)
Difficulty managing stress/time during exams	Yes	389 (79.2)
	No	102 (20.8)
Help-seeking behavior when facing problems	Yes	193 (39.3)
	No	157 (32.0)
	No, but I would like to	141 (28.7)
Primary source of help	Friends	187 (38.0)
	Family	167 (34.0)
	Do not seek help	69 (14.0)
	Psychiatrist	10 (2.0)
	Social counselor	5 (1.0)
	Dorm supervisor	0 (0.0)
Tried counseling inside University of Sharjah (UOS)	Yes	50 (10.2)
	No	441 (89.8)
Tried counseling outside UOS	Yes	75 (15.3)
	No	416 (84.7)
Preferred mode of counseling	Face-to-face	398 (81.1)
	Online	93 (18.9)
Preferred counseling mode	Individual	445 (90.6)
	Group	46 (9.4)
Preferred method of contact	Face-to-face visit	254 (51.7)
	Email	136 (27.7)
	Phone call	101 (20.6)

Factors associated with stress, time management, and counseling utilization

A statistically significant association was observed between gender and difficulty in managing stress and time, with a higher proportion of female students reporting difficulty compared to male students, 302 (85.3%) vs 87 (63.5%) (χ² = 5.64, p = 0.018). Female students had higher odds of reporting stress and time-management difficulty than male students (OR = 3.33, 95% CI: 2.08-5.33). Similarly, ethnicity was significantly associated with stress during examination periods, with Emirati students reporting higher levels of difficulty than non-Emirati students, 131 (84.5%) vs 258 (76.8%) (χ² test, p = 0.050). Stress levels during examination periods were similar across different academic disciplines, and the association was not statistically significant (χ² test, p = 0.34).

Regarding difficulty in finding a suitable study method, a higher proportion of female students reported difficulty compared to male students, 249 (70.3%) vs 81 (59.1%) (χ² test, p = 0.018). In relation to help-seeking behavior and seeking counseling services, 59 (17.9%) of students who reported difficulty sought counseling services outside the university, compared to 16 (9.9%) of those without difficulty; however, this association was not statistically significant (χ² test, p = 0.22).

Among students who had not previously used counseling services, poor stress and time management were significantly associated with willingness to seek counseling, with 143 (36.8%) of students reporting willingness, compared to 246 (63.2%) reporting unwillingness (χ² test, p = 0.024). Similarly, difficulty in managing stress and time was associated with utilization of counseling services at UOS, as only 34 (8.7%) of students reporting difficulty sought counseling, while 355 (91.3%) did not utilize these services (χ² = 4.26, p = 0.039). Students reporting stress and time-management difficulty had lower odds of utilizing UOS counseling services compared with those without difficulty (OR = 0.51, 95% CI: 0.27-0.97).

Students’ academic majors were significantly associated with concern about confidentiality when considering counseling services (χ² test, p = 0.033). Students in medical and health sciences programs reported the highest level of concern, 176 (71%), followed by those in engineering, computing & informatics, and science programs, 87 (61.7%), and students in other colleges (Islamic studies, fine arts, communication, law, and business programs), 59 (57.8%). Awareness of the counseling department location across academic majors showed that 129 (51.8%) of medical/health sciences students, 42 (29.8%) of engineering/science/computing students, and 19 (18.4%) of students from other colleges reported being unaware of the department; however, this association was not statistically significant (χ² test, p = 0.32) (Table 4).

**Table 4:** Association between student characteristics and stress, study difficulty, and counseling-related outcomes (N = 491).

Variable	Category	n	Outcome Measure	Yes n (%)	No n (%)	χ²	p-value
Stress Status	Female	354	Stressed	302 (85.3)	52 (14.7)	5.637	0.018
	Male	137	Stressed	87 (63.5)	50 (36.5)		
	Emirati	155	Stressed	131 (84.5)	24 (15.5)	3.851	0.050
	Non-Emirati	336	Stressed	258 (76.8)	78 (23.2)		
	Medical	248	Stressed	203 (81.9)	45 (18.1)	2.105	0.34
	Eng/Science/Comp	141	Stressed	108 (76.6)	33 (23.4)		
	Other Colleges	102	Stressed	78 (76.5)	24 (23.5)		
Difficulty Finding Study Method	Female	354	Difficulty	249 (70.3)	105 (29.7)	5.637	0.018
	Male	137	Difficulty	81 (59.1)	56 (40.9)		
Sought Help Outside the University of Sharjah (UOS)	No Difficulty	161	Sought Help	16 (9.9)	146 (90.1)	5.272	0.22
	With Difficulty	330	Sought Help	59 (17.9)	271 (82.1)		
Seeking Counseling at UOS	Stress/Time Difficulty: Yes	389	Seeking	34 (8.7)	355 (91.3)	4.263	0.039
	No	102	Seeking	16 (15.7)	86 (84.3)		
Willingness to Seek Help	Poor Stress Management: Yes	389	Willing	143 (36.8)	246 (63.2)	5.090	0.024
	No	102	Willing	50 (49.0)	52 (51.0)		
Confidentiality Concern	Medical Majors & Health Sciences	248	Concern	176 (71.0)	72 (29.0)	6.833	0.033
	Eng/Science/Comp	141	Concern	87 (61.7)	54 (38.3)		
	Other Colleges	102	Concern	59 (57.8)	43 (42.2)		
Awareness of UOS Counseling	Medical Majors & Health Sciences	248	Unaware	129 (51.8)	119 (48.2)	6.868	0.032
	Eng/Science/Comp	141	Unaware	42 (29.8)	99 (70.2)		
	Other Colleges	102	Unaware	19 (18.4)	83 (81.6)		

## Discussion

This cross-sectional study examined awareness, attitudes, and practices related to the utilization of university counseling services among students at UOS. The findings reveal a clear mismatch between students’ mental health needs and their engagement with available counseling services, despite increasing recognition of the importance of university psychological counseling in supporting student well-being and academic success.

Awareness and knowledge of counseling services

A key finding of this study was the limited awareness of the availability of counseling services, with more than half of the respondents reporting that they were unaware that such services were offered by the university. This finding is consistent with previous research conducted in the UAE. In a study among Emirati college students, Al-Darmaki reported that a substantial proportion of students were unaware of the counseling services available at their institutions, including consultation and psychological testing services [13]. Similar gaps in awareness have also been reported among university students in the United States, where a lack of knowledge about available mental health services has been identified as a major contributor to underutilization [14].

In this study, students most commonly reported friends as their primary source of information about counseling services rather than formal university communication channels. This suggests that institutional outreach strategies may not be sufficiently visible or effective. Reliance on informal information sources has been widely documented in the literature, with studies from countries such as Australia, the United States, the United Kingdom, Canada, and Turkey highlighting the central role of peers in shaping awareness and attitudes toward mental health help-seeking [4,15].

Given this reliance on peers, universities may need more proactive, multi-channel promotion of counseling services (e.g., orientation briefings, learning management systems, targeted email/SMS, and visible faculty signposting) to improve mental health literacy and reduce missed opportunities for early support. Evidence from college settings suggests that service use can increase over time with expanded outreach and capacity, yet a substantial proportion of students with clinically significant symptoms still do not receive care, indicating a persistent unmet need [16,17].

Psychological stress and help-seeking behavior

High levels of psychological and academic stress were evident among students, with the majority reporting difficulty managing stress and time during examination periods. This aligns with international evidence demonstrating that university students experience high levels of psychological distress, and that a large proportion of those in need do not access formal mental health services [4,14].

Large multicountry data also indicate that mental disorders are common in university populations, reinforcing the importance of accessible, campus-based support systems for early identification and treatment [18].

Despite this high burden, help-seeking behavior remained limited, with most students preferring to rely on friends or family rather than professional services. Although stress- and time-management difficulties were statistically associated with counseling utilization, the direction of association indicated lower utilization among students reporting higher stress, highlighting a gap between need and service uptake. This pattern reflects findings from prior UAE-based research, which indicate that cultural norms emphasizing privacy and family-based coping may discourage seeking professional psychological help [2]. Gender differences were also observed, with female students reporting greater difficulty managing stress and academic demands, a finding consistent with previous studies among university populations [4].

Barriers to counseling utilization

Stigma and confidentiality concerns emerged as key barriers to counseling utilization. In this study, many students expressed concern about confidentiality when considering counseling services, particularly those enrolled in health sciences programs. Similar concerns have been reported in the UAE and across other Arab populations, such as Saudi Arabia, where stigma surrounding mental health remains a significant deterrent to professional help-seeking [2,19]. These findings underscore the importance of addressing sociocultural perceptions of mental health and reinforcing trust in confidentiality safeguards when promoting counseling services within universities.

A systematic review has shown that stigma has a negative effect on help-seeking, suggesting that reducing stigma and normalizing care-seeking should be core components of university mental health strategies [20].

Practical barriers, such as perceived distance and limited accessibility of counseling services, were also identified in the present study. Similar challenges have been reported in a large cohort of university students in Italy, where counseling services were found to be underutilized when they were perceived as unclear, inconvenient, or insufficiently aligned with students’ expectations, despite being formally available [21]. These findings reinforce the importance of addressing both structural barriers and students’ perceptions of counseling services to improve service uptake.

Preferences for counseling delivery

Students demonstrated a strong preference for face-to-face and individual counseling sessions. This preference is consistent with findings from studies examining attitudes toward psychotherapy among university and medical students, which emphasize the importance of personal interaction and trust in the therapeutic process [22]. These findings suggest that, while digital mental health solutions may serve as useful adjuncts, in-person counseling remains a central component of effective university mental health services.

At UOS, counseling services are delivered through multiple modalities, including individual and group counseling sessions, provided both in person and via online platforms. The availability of these delivery options indicates that students’ strong preference for face-to-face and individual counseling observed in this study likely reflects attitudinal and cultural preferences rather than limitations in service availability.

Implications for university mental health services

The findings of this study suggest that underutilization of counseling services is associated with factors such as limited awareness, perceived stigma, confidentiality concerns, and accessibility barriers, rather than an absence of perceived need. Universities may benefit from implementing structured awareness campaigns, strengthening faculty and peer involvement in mental health promotion, and clearly communicating confidentiality policies. Tailoring counseling services to align with students’ expectations and cultural context may enhance engagement and support both academic success and psychological well-being.

Limitations

The use of convenience sampling and self-reported data may limit generalizability and introduce response bias. As the survey was distributed primarily through online platforms, participation bias may also have occurred, with students who are more digitally active or experiencing higher levels of distress potentially overrepresented in the sample. In addition, the study sample demonstrated a marked gender imbalance, with female students comprising 72.1% of respondents, which may have influenced observed patterns of stress and help-seeking behavior.

Furthermore, the questionnaire did not incorporate standardized, validated measures of psychological distress (e.g., PHQ-9 or GAD-7), limiting the ability to assess symptom severity and compare findings directly with existing literature. Additionally, the questionnaire was newly developed and lacked formal psychometric validation, and, because most constructs were measured using single items, internal consistency reliability could not be assessed.

Although psychiatric treatment was specified as an exclusion criterion, treatment status was not assessed, and no exclusions were applied; therefore, its potential influence on awareness or utilization estimates cannot be determined. In addition, only bivariate analyses were conducted; therefore, independent predictors of counseling utilization could not be determined, and multiple comparisons, without adjustment, may increase the risk of Type I error.

Finally, as the study was conducted at a single institution using a cross-sectional design, causal inferences cannot be made, and findings may not be fully representative of all university students in the UAE.

Future directions

Future research should incorporate qualitative approaches to explore students’ perceptions and concerns in greater depth, particularly regarding stigma and confidentiality. Longitudinal studies assessing the impact of targeted interventions on awareness and service utilization would further inform institutional strategies aimed at promoting student mental health.

## Conclusions

This study highlights a mismatch between the high prevalence of reported academic stress among students at UOS and the low utilization of available university counseling services. Despite the presence of counseling resources, 62.5% of students were unaware of these services, and 65.6% reported confidentiality concerns, indicating persistent gaps in awareness and access. The findings suggest that informational, perceptual, and structural factors may be associated with lower help-seeking behavior within this student population. Overall, this study provides institution-specific and exploratory insight into student engagement with counseling services and underscores the importance of strengthening the connection between student needs and available mental health support within the university setting.

## Appendices

Appendix A

**Table 5:** English version of the questionnaire. This questionnaire was developed by the authors of this study after reviewing the literature.

Section Name	Item Number & Question Type	Questions/Instructions	Choices
Demographics	1. Single choice	Are you a University of Sharjah (UOS) student?	Yes
			No
	2. Single choice	Age	≤17 years
			18-24 years
			≥25 years
	3. Single choice	Ethnicity	Emirati
			Other Arab country
			Non-Arab
	4. Single choice	Gender	Male
			Female
	5. Single choice	College	Sharia and Islamic Studies
			Arts, Humanities and Social Sciences
			Business Administration
			Engineering
			Health Sciences
			Law
			Fine Arts and Design
			Communication
			Medicine
			Dental Medicine
			Pharmacy
			Sciences
			Graduate Studies
			Computing and Informatics
	6. Single choice	Program	Higher Diploma
			Undergraduate
			Master’s
			PhD
	7. Single choice	Do you work?	Yes
			No
Awareness and Knowledge of University Counselling Services	8. Single choice	Are you aware that UOS offers counselling services?	Yes
			No
	9. Multiple choice	How did you find out about the counselling department at UOS?	UOS website
			Friends
			Social media
			Faculty/staff
			I am not aware there is a counselling department at UOS
	10. Single choice	Are you aware of the location and contact details of the counselling department?	Yes
			No
	11. Single choice	Is confidentiality a concern if you seek help from the UOS counselling department?	Yes
			No
	12. Multiple choice	Which services do you think are provided by the counselling department?	Group therapy
			Academic advice
			Family counselling
			Advice for students facing issues
			Segregated male and female sections
			Medication prescription
			Referral to specialist
Performance & Academic Stress	13. Single choice	Which of the following is your top priority?	Studies
			Mental health
			Enhancing social skills
			Sports
			Extracurricular activities
			Friends and family
	14. Single choice	Are you satisfied with your last semester GPA?	Yes
			No
	15. Single choice	Are you struggling to find a suitable study method?	Yes
			No
	16. Single choice	Are you struggling with managing time and stress during exams?	Yes
			No
Attitudes, Help-Seeking Behaviour, and Utilization of Counselling Services	17. Single choice	Do you seek help from others when facing a problem?	Yes
			No
			No, but I would like to
	18. Multiple choice	Who do you turn to for help when facing a problem?	Friends
			Family
			Psychiatrist
			Mutawaa (religious healer)
			Social counsellor
			Senior students
			Faculty/staff
			Dorm supervisor
			I do not seek help
	19. Likert scale	In your opinion, how important is it to contact the counselling department when facing a problem?	Extremely
			Moderately
			Slightly
			Not at all
	20. Multiple choice	Which reasons may prevent you from seeking UOS counselling services?	Bad prior experience
			Do not know location
			Department too far
			Do not know how to contact
			Confidentiality concerns
			Dislike sharing personal information
			Do not believe counselling is effective
			Do not want others to know
			Did not know department exists
			Nothing is stopping me
	21. Single choice	Have you used UOS counselling services?	Yes
			No
	22. Single choice	Have you used counselling services outside UOS?	Yes
			No
	23. Single choice	Counselling preference (mode)	Online
			Face-to-face
	24. Single choice	Counselling preference (format)	Individual
			Group
	25. Multiple choice	Preferred method of contact	Phone call
			Email
			Visiting counselling department

**Table 6:** Arabic version of the questionnaire.

الخيارات	السؤال / التعليمات	رقم البند ونوع السؤال	اسم القسم
نعم	هل أنت طالب/طالبة في جامعة الشارقة؟	1. اختيار واحد	البيانات الديموغرافية
لا			
≤17 سنة	العمر	2. اختيار واحد	
18–24 سنة			
≥25 سنة			
إماراتي	العِرق / الأصل	3. اختيار واحد	
دولة عربية أخرى			
غير عربي			
ذكر	الجنس	4. اختيار واحد	
أنثى			
الشريعة والدراسات الإسلامية	الكلية	5. اختيار واحد	
الآداب والعلوم الإنسانية والاجتماعية			
إدارة الأعمال			
الهندسة			
العلوم الصحية			
القانون			
الفنون الجميلة والتصميم			
الاتصال			
الطب			
طب الأسنان			
الصيدلة			
العلوم			
الدراسات العليا			
الحوسبة والمعلوماتية			
الدبلوم العالي	البرنامج	6. اختيار واحد	
البكالوريوس			
الماجستير			
الدكتوراه			
نعم	هل تعمل؟	7. اختيار واحد	
لا			
نعم	هل تعلم أن جامعة الشارقة تقدم خدمات إرشاد؟	8. اختيار واحد	الوعي والمعرفة بخدمات الإرشاد الجامعي
لا			
موقع الجامعة	كيف عرفت عن قسم الإرشاد في جامعة الشارقة؟	9. اختيار متعدد	
الأصدقاء			
وسائل التواصل الاجتماعي			
أعضاء هيئة التدريس / الموظفون			
لا أعلم بوجود قسم إرشاد			
نعم	هل تعرف موقع وبيانات التواصل الخاصة بقسم الإرشاد؟	10. اختيار واحد	
لا			
نعم	هل تُعد السرية مصدر قلق لك إذا طلبت المساعدة من قسم الإرشاد؟	11. اختيار واحد	
لا			
العلاج الجماعي	ما الخدمات التي تعتقد أن قسم الإرشاد يقدمها؟	12. اختيار متعدد	
الإرشاد الأكاديمي			
الإرشاد الأسري			
تقديم المشورة للطلبة الذين يواجهون مشكلات			
أقسام منفصلة للذكور والإناث			
وصف الأدوية			
الإحالة إلى أخصائي			
الدراسة	أي مما يلي يمثل أولويتك القصوى؟	13. اختيار واحد	الأداء والضغط الأكاديمي
الصحة النفسية			
تطوير المهارات الاجتماعية			
الرياضة			
الأنشطة اللامنهجية			
الأصدقاء والعائلة			
نعم	هل أنت راضٍ/راضية عن معدلك في الفصل الماضي؟	14. اختيار واحد	
لا			
نعم	هل تواجه صعوبة في إيجاد طريقة مناسبة للمذاكرة؟	15. اختيار واحد	
لا			
نعم	هل تواجه صعوبة في إدارة الوقت والضغط أثناء الامتحانات؟	16. اختيار واحد	
لا			
نعم	هل تطلب المساعدة من الآخرين عند مواجهة مشكلة؟	17. اختيار واحد	الاتجاهات وسلوك طلب المساعدة واستخدام خدمات الإرشاد
لا			
لا، ولكن أود ذلك			
الأصدقاء	إلى من تلجأ عند مواجهة مشكلة؟	18. اختيار متعدد	
العائلة			
طبيب نفسي			
مطوّع (معالج ديني)			
مرشد اجتماعي			
طلاب أكبر سناً			
أعضاء هيئة التدريس / الموظفون			
مشرف السكن			
لا أطلب المساعدة			
مهم جداً	برأيك، ما مدى أهمية التواصل مع قسم الإرشاد عند مواجهة مشكلة؟	19. مقياس ليكرت	
مهم بدرجة متوسطة			
مهم بدرجة قليلة			
غير مهم إطلاقاً			
تجربة سابقة سيئة	ما الأسباب التي قد تمنعك من طلب خدمات الإرشاد في جامعة الشارقة؟	20. اختيار متعدد	
لا أعرف الموقع			
القسم بعيد جداً			
لا أعرف كيفية التواصل			
مخاوف تتعلق بالسرية			
لا أحب مشاركة معلومات شخصية			
لا أؤمن بفعالية الإرشاد			
لا أريد أن يعلم الآخرون			
لم أكن أعلم بوجود القسم			
لا شيء يمنعني			
نعم	هل استخدمت خدمات الإرشاد في جامعة الشارقة؟	21. اختيار واحد	
لا			
نعم	هل استخدمت خدمات إرشاد خارج جامعة الشارقة؟	22. اختيار واحد	
لا			
عن بُعد	تفضيل الإرشاد (طريقة التواصل)	23. اختيار واحد	
حضوري			
فردي	تفضيل الإرشاد (الصيغة)	24. اختيار واحد	
جماعي			
مكالمة هاتفية	طريقة التواصل المفضلة	25. اختيار متعدد	
بريد إلكتروني			
زيارة قسم الإرشاد			
